# New aspects of amino acid metabolism in cancer

**DOI:** 10.1038/s41416-019-0620-5

**Published:** 2019-12-10

**Authors:** Lisa Vettore, Rebecca L. Westbrook, Daniel A. Tennant

**Affiliations:** Institute of Metabolism and Systems Research, University of Birmingham, Edgbaston, Birmingham, UK

**Keywords:** Cancer metabolism, Cancer microenvironment

## Abstract

An abundant supply of amino acids is important for cancers to sustain their proliferative drive. Alongside their direct role as substrates for protein synthesis, they can have roles in energy generation, driving the synthesis of nucleosides and maintenance of cellular redox homoeostasis. As cancer cells exist within a complex and often nutrient-poor microenvironment, they sometimes exist as part of a metabolic community, forming relationships that can be both symbiotic and parasitic. Indeed, this is particularly evident in cancers that are auxotrophic for particular amino acids. This review discusses the stromal/cancer cell relationship, by using examples to illustrate a number of different ways in which cancer cells can rely on and contribute to their microenvironment – both as a stable network and in response to therapy. In addition, it examines situations when amino acid synthesis is driven through metabolic coupling to other reactions, and synthesis is in excess of the cancer cell’s proliferative demand. Finally, it highlights the understudied area of non-proteinogenic amino acids in cancer metabolism and their potential role.

## Background

The major function of amino acids in mammalian cells is as substrates for new protein synthesis. There is therefore a significant demand for them in the proliferating cells within a tumour. Those that are essential – defined as those whose carbon skeletons cannot be synthesised by the cell, and include leucine, tryptophan and histidine – are required in significant amounts from both the diet and the intestinal microbiota. Non-essential amino acids can also be synthesised from endogenous sources, providing more flexibility for the cancer cell to ensure an adequate supply. While the definition of non-essential and essential is maintained in tumours at a system-wide level, this classification does not tell the whole story about how cancer cells balance supply and demand within a tumour. Studies over the past few decades have shed considerable light on how transport systems, stromal cells, gene silencing and redox homoeostasis can all play a role in how cancer cells maintain an adequate supply of amino acids to fulfil their proliferative drive. Similarly, they have also suggested novel means of targeting the supply of different amino acids to a cancer cell, with the potential for novel therapeutic interventions to block tumour growth in the future. This review will examine aspects of the control of amino acid metabolism, including when ‘non-essential’ amino acids become limiting for tumour growth, how the stroma is often utilised to provide these nutrients and conditions in which the synthetic pathways are regulated by other factors, sometimes producing amino acids in excess of tumour demand. Finally, we briefly discuss the emerging interest in non-proteinogenic amino acids in tumour metabolism.

## Non-essential yet required?

A number of cancers have been found that are auxotrophic (i.e. depend on exogenous sources) for non-essential amino acids. In a number of cases this is through simple loss of expression of an enzyme involved in its synthesis through direct mutation or silencing.^1,2^ Three such examples of this are the synthesis of arginine, asparagine and glutamine. In most cell types, arginine can be synthesised through the activity of argininosuccinate synthetase (ASS1) and argininosuccinate lyase (ASL), their combined activities transferring the amino group of aspartate to convert citrulline into arginine. It has been shown that ASS1 is not expressed in a number of different malignancies including some melanoma, prostate cancer, hepatocellular carcinoma, mesothelioma and bladder cancers.^3–5^ Given that this loss of ASS1 expression, and therefore the ability to synthesise arginine de novo is specific to malignant cells, trials of the non-mammalian enzyme, arginine deiminase (ADI), as a potential therapy to selectively kill the cancer cells have been performed and are ongoing.^6,7^

Silencing of asparagine synthetase (ASNS), the enzyme that uses the amide group from glutamine to synthesise asparagine from aspartate has also been shown as a cause of cancer-specific auxotrophy. It has been known since the 1970s that the response of paediatric acute lymphoblastic leukaemia (ALL) to therapy was inversely correlated with asparagine synthesis. Indeed, this observation led to the use of bacterial asparaginase (ASNase) to cure the majority of children with this cancer, either as single agent or as combination therapy,^8^ making it one of the most successful forms of metabolism-targeted therapy.

A further, less well-described auxotrophy is for glutamine, through loss or downregulation of glutamine synthetase (GS), which has been described in multiple myeloma, ovarian cancer and oligodendroglioma cells.^9–11^ GS synthesises glutamine from glutamate and NH_4_^+^, which has previously been shown to be important for continued tumour proliferation, particularly when glutamine may be limiting.^12^ The plasma membrane glutamine transporters may therefore represent potential therapeutic targets for tumours with GS deficiency. ASNase treatment, which depletes plasma glutamine in addition to asparagine, is also likely to show some efficacy in tumours lacking GS, although alternative means of depleting plasma glutamine by using phenylacetate have previously been trialled, albeit without stratification of patients for GS expression status.^13,14^ Interestingly, stromal cell expression of GS may also be important for cancer growth, suggesting that targeting this mechanism may indirectly reduce tumour cell proliferation.^15^

It is particularly interesting that these examples of tumour-associated auxotrophy are centred around two reactions that utilise aspartate to synthesise other amino acids, both directly (ASNS and ASS1) and indirectly (GS, through regeneration of glutamine required for ASNS activity). It is becoming clear that aspartate is a significant metabolic hub, and a major product of the oxidative glutaminolysis pathway. Aspartate is a required substrate for other anabolic pathways, including the synthesis of purines (both directly within the base carbon skeleton and amination of IMP to form AMP) and pyrimidines. It is an appealing hypothesis that aspartate may be limiting in some tumours,^16^ and that cancer cells selectively prioritise synthesis of nucleotides over asparagine and arginine, given that the amino acids may be more easily available within the microenvironment than substrates for the nucleotide salvage pathways. Indeed, this can be further highlighted by the recently reported depletion of aspartate observed in some rare tumour types driven by mutations in the metabolic enzyme, and a key component of the glutaminolytic pathway, succinate dehydrogenase.^17,18^

However, it is important to note that resistance to asparaginase and arginase therapy has been observed without re-expression of ASS1 or ASNS. One potential short-term mechanism of resistance has been suggested as autophagy, by resupplying intracellular amino acids during therapies that deplete exogenous sources.^19,20^ However, this mechanism is not conducive to long-term survival in a proliferating tumour – a more dependable supply in the long term is likely to come from tumour-associated stromal cells.^21^ In the case of ASNase treatment of ALL, amino acid supply from mesenchymal-derived stromal cells (asparagine) and adipocytes (glutamine) has been reported, reducing the efficacy of therapeutic depletion of asparagine in the peripheral blood.^21,22^ This paradigm does not appear to end with those tumour cells that require specific amino acids for growth, but forms part of a continuum in which stromal and tumour cells swap nutrients including amino acids as part of a wider tumoural metabolic network.

## The metabolic relationship between tumour and stroma: symbiotic or parasitic?

The trade in metabolites between stromal cells and the cancer cells themselves is becoming increasingly seen as an important facet of tumour metabolism, and often involves the transport of amino acids. One recently reported bidirectional relationship is between cancer-associated fibroblasts (CAFs) and cancer cells. In this model, it was shown that CAFs provide aspartate to cancer cells, which is taken up via the transporter SLC1A3 (also known as the excitatory amino acid transporter 1 [EAAT1]) to support nucleotide biosynthesis, while tumour cells reciprocate with glutamine-derived glutamate taken up by the CAFs through the same transporter.^23^ The nature of the transport(s) responsible for efflux of these two amino acids from cancer and stromal cells is not yet clear. Interestingly, the expression of SLC1A3, a transporter originally described as being central in regulating extracellular glutamate levels in synapses, was more recently described as being regulated by p53 in cancer cells, providing the cells with aspartate during glutamine deprivation.^24^ This metabolic relationship has significant benefits for the cancer cell and may lead to less reliance on the oxidative TCA cycle for proliferation, given that glutamate is swapped for aspartate rather than oxidatively metabolised to synthesise it.^25,26^ This may have the effect of changing the profile of nutrients that are incorporated into the TCA cycle, reducing oxygen consumption by the cancer cells. The knock-on effect would be that the increased conversion of glutamate into aspartate by the stromal cells would probably have the opposite effect on them, i.e. by increasing their requirement to respire to oxidise NAD^+^ (see Fig. 2), thereby making them more sensitive to complex I inhibition, such as by metformin.

**Fig. 1:**
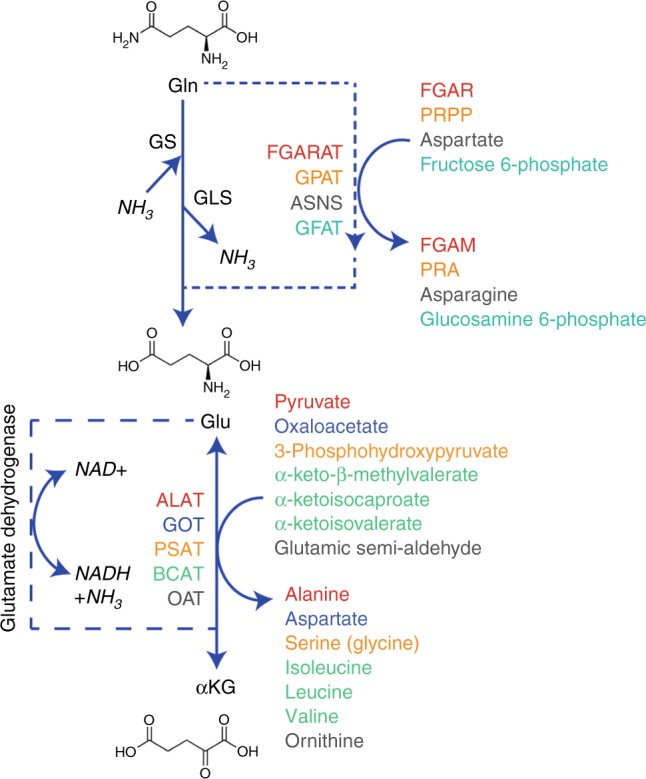
Metabolism of glutamine. The amido group of glutamine is involved in relatively few reactions in addition to deamidation, some major examples are indicated. Transamination, for which only some representative reactions are shown, involves a number of 2-oxoacids that can be reversibly converted into the amino acid. Colours are used in both sets of reactions to indicate the enzyme (left) responsible for the reaction (right). On the left are the reactions that require, or evolve ammonia as part of the metabolism of glutamine to or from α-ketoglutarate. Abbreviations: αKG α-ketoglutarate, ALAT, alanine aminotransferase, ASNS, asparagine synthetase, BCAT, branched-chain aminotransferase, FGAM 5′-phosphoribosyl-N-formylglycinamidine, FGAR 5′-phosphoribosyl-N-formylglycinamide, FGARAT FGAR amidotransferase, GFAT glutamine fructose 6-phosphate amidotransferase, Gln glutamine, GLS glutaminase, Glu glutamate, GPAT glutamine phosphoribosyl pyrophosphate amidotransferase, GOT glutamic-oxaloacetic aminotransferase, GS glutamine synthetase, OAT ornithine aminotransferase, PRA 5′-phosphoribosyl-1-amine, PRPP 5′-phosphoribosyl-1-pyrophosphate, PSAT phosphoserine aminotransferase.

The amido group of glutamine is used to drive an entirely different spectrum of reactions compared with those utilising the amino group, including purine synthesis, asparagine synthesis and O-linked glycosylation (Fig. 1). The importance of the amido nitrogen to drive cancer cell proliferation has recently been described in glioma, where it was shown that glutamine-derived glutamate had two potential fates: amidation within the glioma cells by glutamine synthase (by using free ammonia) to regenerate glutamine, or export for uptake and amidation by astrocytes.^12^ This second system permits secretion of the glutamine by astrocytes and uptake by the glioma cells, effectively permitting the astrocytes to fix ammonia from the microenvironment to be utilised for nucleotide synthesis by the tumour.

The two examples highlighted above show two relationships between cancer and stromal cells: one in which both cells appear to benefit, and another in which the cancer cells take advantage of a physiologically relevant stromal activity. This is also the case when the relationship between the cell providing the amino acid and the cancer cell is physically more distant. For example, the process of tumour-associated cachexia, in which muscles and adipose tissue are progressively catabolised, is often observed in late-stage patients as their tumours continue to require nutrients in excess of what is contained in the diet.

An alternative means by which cancer cells can take up amino acids, but this time en masse, comes in the form of a process known as macropinocytosis. Described as ‘cell-drinking’, it is a process in which cells take up gulps of their microenvironment, known in a number of cell types under non-pathological conditions.^27^ Although first described in malignant cells in 1937,^28^ and then identified as being driven by oncogenic RAS in 1986,^29^ it was not until 2013 that a metabolic function was ascribed to macropinocytosis in cancer cells, when it was shown to result in uptake of whole proteins in pancreatic ductal adenocarcinoma (PDAC) cells.^30^ These proteins were catabolised through the autophagic machinery by the cancer cells, providing free amino acids for new protein synthesis, or catabolism to produce ATP.^30,31^ This process allows the cancer cells to survive in relatively nutrient-poor environments, taking up a number of different macromolecules derived from both the periphery (probably accumulating in oedema) and necrotic areas of the tumour. As well as being activated by other oncogenic signalling pathways, such as in those tumours driven by loss of Phosphatase and tensin homolog (PTEN) or aberrant WNT signalling,^32,33^ macropinocytosis can also be induced by the metabolic microenvironment of the tumour, as shown by macropinocytosis of albumin-associated free fatty acids by colorectal cancer cells through G protein-coupled receptor 120 (GPR120).^34^ Given that this process allows a tumour cell to continue to proliferate in the absence of abundant exogenous nutrients, it may play a particularly important role in hypoxic tumour regions, where not only are exogenous nutrients often limiting, but also where necrotic areas are juxtaposed to the malignant cells.

There is also evidence that cancers utilise amino acids derived from the extracellular matrix to support metabolism, in particular proline for ATP generation.^35,36^ In one study, the catabolism and uptake of collagen fragments were found to support PDAC tumour survival, by using macropinocytosis amongst other mechanisms when nutrient-deprived.^36^ Another study in breast cancer showed that in metastatic models of breast cancer, proline uptake and degradation supported survival.^35^ In this latter model, it is likely that plasma concentrations of proline can support this metabolic phenotype (~170 µM^37^), although digestion of the extracellular matrix (ECM) in the target tissue may also be important upon extravasation and colonisation in the absence of appropriate nutrient supply.

Tumours have therefore found means of utilising diverse mechanisms to provide themselves a supply of the amino acids that they require for malignant progression. It remains the case that many of these mechanisms are probable targets for novel therapy. However, given that a number of them are based upon non-pathological processes by which cells form a healthy metabolic community, care will need to be taken to avoid on-target side effects.

## Metabolic coupling in the regulation of amino acid synthetic pathways

**Fig. 2:**
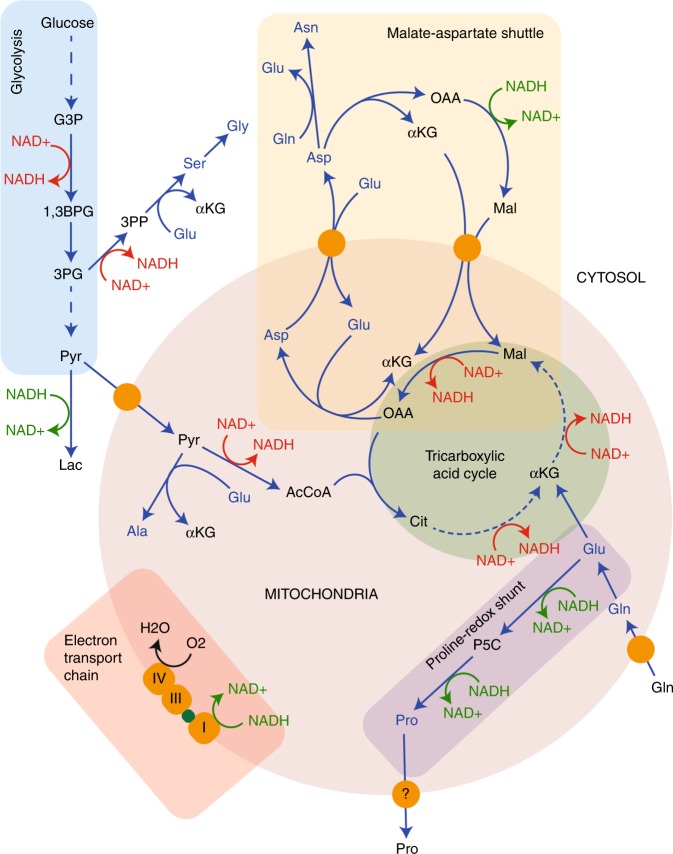
Interplay between amino acid metabolism and redox homoeostasis. The synthesis and catabolism of amino acids is interwoven into the redox homoeostasis of the cell. The malate–aspartate shuttle, as well as moving NADH between the cytosol and the mitochondrial matrix, also moves the amino acids glutamate and aspartate between the two compartments, and is functionally connected to the TCA cycle. When aspartate is removed from this cycle to synthesise asparagine, arginine or nucleosides, this would disrupt the cycle, requiring additional carbon input. NADH oxidation reactions are shown in green, while NAD^+^ reduction is shown in red, indicative of the connectivity of this network. Amino acids are represented in blue, while proteins (transporters and electron carriers) are in orange. Finally, the subnetworks illustrated are outlined in blue (glycolysis), pink (electron transport chain), mauve (proline-redox shunt), green (TCA cycle) and cream (malate–aspartate shuttle). Abbreviations: αKG α-ketoglutarate, 1,3BPG 1,3-bisphosphoglycerate, 3PG 3-phosphoglycerate, 3PP 3-phosphopyruvate, AcCoA acetyl CoA, Ala alanine, Asn asparagine, Asp aspartate, Cit citrate, G3P glyceraldehyde 3-phosphate, Gln glutamine, Glu glutamate, Gly glycine, Lac lactate, Mal malate, NAD^+^ nicotinamide adenine dinucleotide, NADH reduced NAD^+^, OAA oxaloacetate, P5C pyrroline 5-carboxylate, Pro proline, Pyr pyruvate, Ser serine.

In common with most enzymatic reactions, non-essential amino acid synthesis requires more than one substrate and results in more than one product. Most often, this reaction is a simple transamination by using glutamate as the amino donor, resulting in the generation of α-ketoglutarate (Fig. 1). The direction of these reversible reactions depends on the local concentrations of the substrates and products as they are close to being energetically neutral. The relative concentrations of glutamate and α-ketoglutarate within the cytoplasm and mitochondria are therefore critical in determining which amino acid is made or catabolised, and how this reaction influences the wider metabolic network. A good example of this is the synthesis of aspartate. Within the mitochondrion, oxaloacetate (OAA) is most often a product of oxidative TCA cycle activity (Fig. 2), while glutamate is produced by mitochondrial glutaminase. Coupling these two reactions together in this compartment maintains flux of α-ketoglutarate into the TCA cycle, while removing OAA to permit continued activity, producing reducing potential to generate ATP.^38^ In this way, two metabolites central to proliferation – ATP and aspartate – are synthesised in parallel.^25,26^ These links go further, given that aspartate, glutamate, α-ketoglutarate and malate are functionally coupled through the malate–aspartate shuttle, which is important for moving reducing potential between the matrix and the cytosol (Fig. 2).

Alanine is another amino acid, the synthesis of which (from pyruvate) is thought to lie mainly in the mitochondrial matrix.^39^ The alanine aminotransferase (ALAT) therefore competes for mitochondrial pyruvate with pyruvate dehydrogenase (PDH), which oxidatively decarboxylates to form acetyl-CoA (Fig. 2). It would therefore be expected that in conditions where PDH activity is lower, which includes hypoxia, increased alanine synthesis may be observed. However, recent evidence suggesting that ALAT activity is important for driving ECM formation through α-ketoglutarate production in metastatic breast cancer cells,^40^ has shed a different light on the role of amino acid synthesis. The data define this pathway as one that can be under regulation by coupled reactions – the synthesis of alanine is regulated under some conditions by a demand for α-ketoglutarate and driven by uptake of pyruvate from the microenvironment.^40^ Under such conditions, the fate of the alanine is not clear but is  probably in excess of what is required for anabolism – this would indicate that it is excreted into the extracellular space and would suggest the potential for it to be taken up by stromal cells, which may secrete the pyruvate required for the reaction themselves. Interestingly, this system demonstrates a reversal of that previously described in PDAC, where the major stromal cell type, the stellate cell, secretes alanine that is taken up by the cancer cells and deaminated to pyruvate as a significant carbon source.^41^ This means that while increasing oxygen consumption in what is a highly hypoxic tumour type – which appears somewhat counterintuitive – it also spares glucose to be used by other cell types or for synthesis of macromolecular substrates, including serine and glycine.

A further example of amino acid synthesis regulated by a coupled metabolic reaction was the recently described synthesis of proline in glioma. Our research group investigated the apparent drivers of the increased production and excretion of proline by cells expressing an oncogenic mutant form of the enzyme, isocitrate dehydrogenase 1 (IDH1), which is regularly observed in grade II and III gliomas and around 10% of grade IV gliomas (glioblastomas).^42,43^ The normal activity of IDH1 involves the NADPH-driven carboxylation of α-ketoglutarate to isocitrate, which is reversible depending on the redox state of the cell. However, the IDH1 mutation observed in gliomas results in a neomorphic activity that reduces α-ketoglutarate to 2-hydroxyglutarate, driven by the oxidation of NADPH.^44^ This has been suggested to perturb the redox homoeostasis of the cell.^45–47^ It has been previously well-documented that proline synthesis may be a means of responding to adverse environmental conditions, including redox stress, in plants.^48^ In mammalian systems, proline synthesis is through two routes: either in the cytosol from ornithine through the activity of ornithine aminotransferase and pyrroline 5-carboxylate reductase 3 (PYCR3, also known as PYCRL), or in the mitochondria from glutamate through either PYCR1 or 2. Each of these reductase reactions is linked to NADPH oxidation –PYCR3 appears to have a higher activity in the presence of NADPH, while PYCR1 and 2 favour NADH.^49^ Interestingly, we showed that cells expressing the oncogenic mutant of IDH1 increased proline synthesis and excretion, but this appeared to be specifically through the increased activity of mitochondrial PYCR1 by using glutamine as the major carbon source.^42^ The fact that proline synthesised in these conditions was excreted suggested that its synthesis may be driven by oxidation of NADH, rather than a demand for proline. Indeed, the reduction of glutamate to proline oxidises two moles of NADH per mole of proline synthesised, making it a very efficient means of responding to an increased demand for NAD^+^ (Fig. 2). Flux along this pathway is likely to be increased under general conditions in which the mitochondrial NADH:NAD^+^ ratio increases, which would include hypoxia and where there are deficiencies in NADH oxidation due to mitochondrial dysfunction. This mechanism is conceptually the same as reduction of cytosolic pyruvate by lactate dehydrogenase to regenerate NAD^+^ in the cytosol, although it may be more efficient in terms of NADH oxidised to carbons exported (2.5/NADH [proline] compared with 3/NADH [lactate]). However, the means of exporting proline from the matrix is currently unclear and this may add additional complexity to the system, as it is likely either to drive increased mitochondrial membrane potential (through proton symport) or require import of another metabolite into the matrix. Interestingly, there is a further potential role for increased proline synthesis in conditions where redox homoeostasis is perturbed. Proline can react with hydroxyl radicals to form moieties such as 4-hydroxyproline and 3-hydroxyproline, although it has been shown not to react with either peroxide or superoxide over shorter timescales. It is worth noting that this may change with longer-term oxidative stress.^50–52^

Lastly, some cytosolic amino acid pools are used to drive the uptake of other amino acids, meaning that a reduction in the cytosolic pool of one amino acid may lead to a deficiency in another seemingly unrelated member of the family. Indeed, the upregulation of amino acid transporters in cancer maintains amino acid pools at a level that supports its malignant hallmarks.^53^ This can be highlighted by a number of examples: the export of glutamine to drive leucine uptake,^54^ asparagine for serine, arginine and histidine^55^ and glutamate for cystine.^56^ Cystine is the oxidised homodimer of cysteine, connected via a disulfide bridge, and is the form of the amino acid found in the extracellular space. It enters the cell via a member of the heteromeric amino acid transporter family, known as the system x_C_^–^ transporter, which consists of the SLC3A2-encoded CD98 protein covalently linked to the SLC7A11-encoded xCT.^57^ The former is required for cell-surface expression, while xCT is responsible for the specific carrier activity. In order to transport cystine into the cell, the transporter antiports glutamate out of the cell, functionally linking these two amino acids, which alongside glycine are critical for the synthesis of the major antioxidant, glutathione.^58,59^ As cellular cysteine availability is the rate-limiting step in glutathione synthesis,^59^ the function of the xCT transporter, and therefore intracellular glutamate concentrations, are critical for the synthesis of this major cellular antioxidant. Accordingly, as glutathione concentrations can determine the response of tumours to therapy, xCT has been suggested as a putative therapeutic target.^57,60,61^ There is significant demand for glutamate within a proliferating cell not only as an amino acid per se, but also from numerous transamination reactions, use of its carbon backbone for the synthesis of other anabolic metabolites and antioxidant synthesis. Interestingly, this suggests that glutamate could become limiting for proliferation in some conditions. It has been suggested that glutamate could be synthesised through the action of glutamate dehydrogenase on cellular α-ketoglutarate, recycling the ammonia produced by glutaminase activity and reducing NAD^+^ in the process (Fig. 1).^62^ Where glutamate is limiting, this reaction may therefore support tumour growth – a pathway shown to be relevant in breast cancer.^62^

Glycine, the third amino acid required for glutathione synthesis, is synthesised alongside serine from the glycolytic intermediate, 3-phosphoglycerate (3PG, Fig. 2) and can also be regulated by a coupled metabolic reaction. While the synthesis of serine requires both the generation of cytosolic NADH and glutamate (Fig. 2), the synthesis of glycine from serine is used to provide methyl groups for both nucleoside synthesis and regeneration of S-adenosylmethionine (SAM), required for methylation of proteins and DNA.^63^ It was also recently suggested that serine and glycine metabolism within cancer cells includes the use of a mitochondrial pathway, which, through the catabolism of serine to formate (which is excreted), generates mitochondrial NADH methyl groups for nucleotide synthesis.^64^ This pathway was since shown to depend on mitochondrial NADH oxidation (and therefore ATP synthesis).^65^ In this way, it represents another means of translocating cytosolic NADH into the mitochondrion, similar to the malate–aspartate shuttle, while also producing one-carbon intermediates for cellular anabolism.

In summary, it is apparent that under certain conditions, the synthesis of amino acids can be used by cancer cells to drive the other products of these reactions – whether to preserve redox homoeostasis, maintain α-ketoglutarate concentrations or for the import of other amino acids.

## Non-proteinogenic amino acids in cancer metabolism

Those amino acids that can be used for protein synthesis are in fact a small subset of the total amino acid pool in the mammalian metabolome. Alongside those that are dimers of proteinogenic amino acids (including cystine and cystathionine), there are those with a β, γ or δ rather than α chiral centres (such as taurine, β-alanine and δ-aminolevulinic acid) and those with a proteinogenic structure that are not utilised in de novo protein synthesis (e.g. N-acetyl-l-glutamate, ornithine and citrulline).

Taurine is either synthesised from methionine (via cysteine) or taken up in the diet. It has several suggested functions, including efficient loading of amino acids onto some mitochondrial tRNAs in free radical scavenging and regulation of cellular osmolarity.^66–68^ It is transported across the plasma membrane by SLC6A6 (TauT), which also transports β-alanine, and by SLC36A1 (PAT1), which transports amino acids such as alanine, glycine and serine, as well as other substrates.^69,70^ There have been reports that taurine levels in vivo are increased in specific tumour types in the brain,^71,72^ and that it may play a role in the induction of cancer cell death.^73^ However, the main role of taurine in either health or disease is as yet unknown.

The metabolism of δ-aminolevulinic acid (5-aminolevulinic acid, 5ALA) is also clearly altered in some tumour types but has a yet-undefined role. 5ALA is the substrate for porphyrin synthesis, which forms the basis of successful responses to photodynamic therapy used to treat some tumour types, and is increasingly used to guide surgery – particularly in patients with glioma.^74^ The transporter for this amino acid is as yet unclear. SLC6A6, SLC6A13 (GAT2), SLC15A1 and SLC15A2 (PEPT1 and 2) have all been suggested, with the likelihood being that this function is tissue-specific.^75–77^ The fact that some tumour types take up and metabolise this amino acid at significantly greater quantities than the surrounding tissue – the basis of its use as a clinical imaging agent – suggests that we understand little of tumour requirements and use for this amino acid.

With these examples, it is clear that many of the non-proteinogenic amino acids have been highly understudied thus far, despite the fact that indications in patients suggest that they may play a role in the biological underpinning of tumours. The lack of investigations is most likely due to their absence in tissue culture medium, despite being present at often significant concentrations in tissues, plasma and cerebrospinal fluid. Indeed, both the recently described plasma-like culture media contain many of these metabolites,^78,79^ indicative of an increasing awareness that our common culture systems do not adequately address many aspects of cancer cell biology, forcing the metabolic network into an aberrantly small space and reducing our ability to efficiently translate novel agents to target cancer metabolism into patients.

## Summary

Investigations thus far into the metabolism of amino acids in cancer has begun to highlight a nuanced network, where in many cases, tracing of the uptake and use of a specific amino acid may not reveal the real functional output of the pathway. Tumours silence expression of genes required for synthesis of some amino acids, producing an auxotrophy that requires metabolic symbiosis with the stroma. However, the stroma is also highly relied upon to provide additional nutrients to support viability and proliferation, with the cancer cells only sometimes providing something to the stromal cells in return. This clearly describes a metabolic network that requires modelling approaches that take into account multiple cell types as well as physiologically relevant media. Indeed, as these media contain ‘plasma-like’ concentrations of proteinogenic amino acids, the direction and magnitude of fluxes observed are more likely to represent those observed in patients, which can therefore be more reproducibly translated into efficacious agents that can eventually improve patient care.

## Data Availability

Not applicable
